# Temporal mapping of derived high-frequency gene variants supports the mosaic nature of the evolution of *Homo sapiens*

**DOI:** 10.1038/s41598-022-13589-0

**Published:** 2022-06-15

**Authors:** Alejandro Andirkó, Juan Moriano, Alessandro Vitriolo, Martin Kuhlwilm, Giuseppe Testa, Cedric Boeckx

**Affiliations:** 1 Universitat de Barcelona, Barcelona, Spain; 2 Universitat de Barcelona Institute of Complex Systems (UBICS), Barcelona, Spain; 3 University of Milan, Milan, Italy; 4 European Institute of Oncology (IEO), Milan, Italy; 5 Human Technopole, Milan, Italy; 6 University of Vienna, Vienna, Austria; 7 Human Evolution and Archaeological Sciences (HEAS), University of Vienna, Vienna, Austria; 8 Catalan Institute for Research and Advanced Studies (ICREA), Catalonia, Spain

**Keywords:** Computational biology and bioinformatics, Evolution

## Abstract

Large-scale estimations of the time of emergence of variants are essential to examine hypotheses concerning human evolution with precision. Using an open repository of genetic variant age estimations, we offer here a temporal evaluation of various evolutionarily relevant datasets, such as *Homo sapiens*-specific variants, high-frequency variants found in genetic windows under positive selection, introgressed variants from extinct human species, as well as putative regulatory variants specific to various brain regions. We find a recurrent bimodal distribution of high-frequency variants, but also evidence for specific enrichments of gene categories in distinct time windows, pointing to different periods of phenotypic changes, resulting in a mosaic. With a temporal classification of genetic mutations in hand, we then applied a machine learning tool to predict what genes have changed more in certain time windows, and which tissues these genes may have impacted more. Overall, we provide a fine-grained temporal mapping of derived variants in *Homo sapiens* that helps to illuminate the intricate evolutionary history of our species.

## Introduction

The past decade has seen a significant shift in our understanding of the evolution of our lineage. We now recognize that anatomical features used as diagnostic for our species (globular neurocranium, small, retracted face, presence of a chin, narrow trunk, to cite only a few of the most salient traits associated with “anatomical modernity”) did not emerge as a package, from a single geographical location, but rather emerged gradually, in a mosaic-like fashion across the entire African continent and quite possibly beyond^1–3^. Likewise, behavioral characteristics once thought to be exclusive of *Homo sapiens* (funerary rituals, parietal art, ‘symbolic’ artefacts, etc.) have recently been attested in some form in closely related (extinct) clades, casting doubt on a simple definition of ‘cognitive/behavioral’ modernity^4^. We have also come to appreciate the extent of repeated (multidirectional) gene flow between *Homo sapiens* and Neanderthals and Denisovans, raising interesting questions about speciation^5–8^. Last, but not least, it is now well established that our species has a long history. Robust genetic analyses^9^ indicate a divergence time between us and other hominins for whom genomes are available of roughly 700kya, leaving perhaps as many as 500ky between then and the earliest fossils displaying a near-complete suite of modern traits (Omo Kibish 1, Herto 1 and 2)^10^. Such a long period of time is likely to contain enough opportunities for multiple rounds of evolutionary modifications. Taken together, these findings render completely implausible simplistic narratives about the ‘modern human condition’ that seek to identify a specific geographical location or genetic mutation that would ‘define’ us^11^.

Genomic analysis of ancient human remains in Africa reveal deep population splits and complex admixture patterns among populations^12–14^. At the same time, reanalysis of fossils in Africa^15^ points to the extended presence of multiple hominins on this continent, together with real possibilities of admixture^16,17^. Lastly, our deeper understanding of other hominins points to derived characteristics in these lineages that make some of our species’ traits more ancestral (less ‘modern’) than previously believed^18^.

In the context of this significant rewriting of our deep history, we decided to explore the temporal structure of an extended catalog of single nucleotide changes found at high frequency (HF $\ge$ 90%) across major modern populations we previously generated on the basis of 3 high-coverage “archaic” genomes^19^, that is, Neanderthal/Denisovan individuals, used as outgroups. This catalog aims to offer a richer picture of molecular events setting us apart from our closest extinct relatives. In order to probe the temporal nature of this data, we took advantage of the Genealogical Estimation of Variant Age (GEVA) tool^20^. GEVA is a coalescence-based method that provides age estimates for over 45 million human variants. GEVA is non-parametric, making no assumptions about demographic history, tree shapes, or selection (for additional details on GEVA, see “Methods”). Our overall objective here is to use the temporal resolution afforded by GEVA to estimate the age of emergence of polymorphic sites, and gain further insights into the complex evolutionary trajectory of our species.

Our analysis reveals a bimodal temporal distribution of modern human derived high-frequency variants and provides insights into milestones of *Homo sapiens* evolution through the investigation of the molecular correlates and the predicted impact of variants across evolutionary-relevant periods. Our chronological atlas allows us to provide a time window estimate of introgression events and evaluate the age of variants associated with signals of positive selection, tissue-specific changes, and specifically an estimate of the age of emergence of (enhancer) regulatory variants associated with different brain regions. Our enrichment analysis uncovers GO-terms unique to specific temporal windows, such as facial and behavioral-related terms for a period (between 300 and 500 k years) preceding the dating of human fossils like that of Jebel Irhoud. Our machine learning-based analyses predicting differential gene expression regulation of mapped variants (through^21^) reveals a trend towards downregulation in brain-related tissues and allowed us to identify variant-associated genes whose differential regulation may specifically affect brain structures such as the cerebellum.

## Results

**Figure 1:**
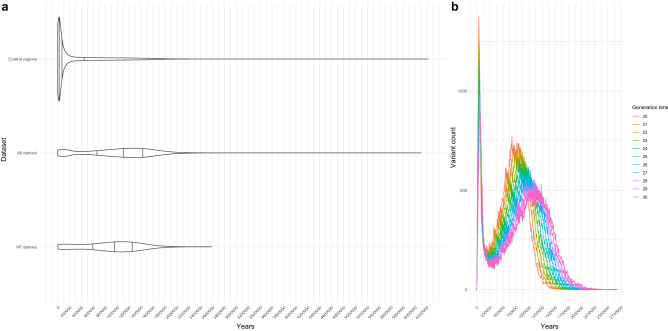
(**a**) Density of distribution of derived *Homo sapiens* alleles over time in an aggregated control set (n = 1000) of random variants across the genome and two sets of derived ones: all derived variants, and those found at high-frequency. Horizontal lines mark distribution quantiles 0.25, 0.5 and 0.75. (**b**) Line plot showing the bimodal distribution of high-frequency variants using different generation times (in the text, we used 29 years, following^62^).

The distribution of derived alleles over time follows a bimodal distribution (Fig. 1a,b; see also Fig. S2 for a more elaborated version), with a global maximum around 40 kya (for complete allele counts, see “Methods”). The two modes of the distribution of HF variants likely correspond to two periods of significance in the evolutionary history of *Homo sapiens*. The more recent peak of HF variants arguably corresponds to the period of population dispersal and replacement following the last major out of Africa event^22,23^, while the older distribution contains the period associated with the divergence between *Homo sapiens* and other *Homo* species^9,24^.

**Figure 2:**
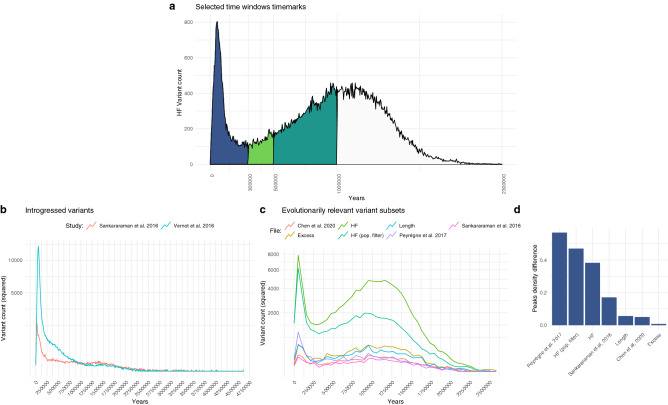
(**a**) Selected temporal windows used in our study to further interrogate the nature and distribution of HF variants. (**b**) Distribution of introgressed alleles over time, as identified by^27,30^. (**c**) Plots of HF variants in datasets relevant to human evolution, including regions under positive selection^29^, regions depleted of archaic introgression^27,28^ and genes showing an excess of HF variants (‘excess’ and ‘length’)^19^. Variant counts in (**a**,**c**,**d**) are squared to aid visualization. (**d**) Kernel density difference between the highest point in the distributions of (**d**) (leftmost peak) and the second, older highest density peak, normalized, in percentage units.

In order to divide the data into smaller temporal clusters for downstream analysis we considered a *k*-means clustering analysis (at $k=3$ and $k=4$, Fig. S1). This clustering method yields a division clear enough to distinguish between “early” and “late” *Homo sapiens* “specimens”^10^, with a protracted period overlapping with the split with other *Homo* species. (The availability of ancient DNA from other hominins would yield a better resolution of that period.) However, we reasoned that such a k-means division is not precise enough to represent key milestones used to test specific time-sensitive hypotheses. For this reason, we adopted a literature-based approach, establishing different cutoffs adapted to the need of each analysis below. Our basic division consisted of three periods (see Fig. 2a): a recent period from the present to 300 thousand years ago (kya), the local minimum, roughly corresponding to the period considered until recently to mark the emergence of *Homo sapiens*^12^; a later period from 300 to 500 kya, the period right before the dating of fossils associated with earlier members of our species such as the Jebel Irhoud fossil^25^ and, incidentally, the critical juncture between the first and second temporal windows when comparing the two *k*-means clustering analyses we performed (Fig. S1); and a third, older period, from 500 kya to 1 million years ago, corresponding to the time of the most recent common ancestor with the Neanderthal and Denisovan lineages^24,26^.

We note that the distribution goes as far back as 2.5 million years ago (see Fig. 1a) in the case of HF variants, and even further back in the case of the derived variants with no HF cutoff. This could be due to our temporal prediction model choice (GEVA clock model, of which GEVA offers three options, as detailed in “Methods”), as changes over time in human recombination rates might affect the timing of older variants^20^, or to the fact that we do not have genomes for older *Homo* species. Some of these very old variants may have been inherited from them and lost further down Neanderthal/Denisovan lineages.

### Variant subset distributions

In an attempt to see if specific subsets of variants clustered in different ways over the inferred time axis, we selected a series of evolutionary relevant sets of data publicly available, such as genome regions depleted of “archaic” introgression (so-called ‘deserts of introgression’)^27,28^, and regions under putative positive selection^29^, and mapped the HF variants from^19^ falling within those regions. We also examined genes that accumulate more HF variants than expected given their length and in comparison to the number of mutations these genes accumulate on the Neanderthal/Denisovan lineages (‘length’ and ‘excess’ lists from^19^—see “Methods”). Finally, we also examined the temporal distribution of introgressed alleles^27,30^. A bimodal distribution is clearly visible in all the subsets except the introgression datasets (Fig. 2b). Introgressed variants peak locally in the more recent period (0–100 kya). The distribution roughly fades after 250 kya, in consonance with the possible timing of introgression events^6,16,28,31^. As a case study, we focused on those introgressed variants associated with phenotypes highlighted in Table 1 of^32^. As shown in Fig. S3, half of the variants cluster around the highest peak, but other variants may have been introduced in earlier instances of gene flow. We caution, though, that multiple (likely) factors, such as gene flow from Eurasians into Africa, or effects of positive selection affecting frequency, influence the distribution of age estimates and make it hard to draw any firm conclusions. We also note that the two introgressed variant counts, derived from the data of^27,30^, follow a significantly different distribution over time ($p<$ 2.2–16, Kolmogorov–Smirnov test) (Fig. 2c).

Finally, we examined the distribution of putatively introgressed variants across populations, focusing on low-frequency variants whose distributions vary when we look at African vs. non-African populations (Fig. S4). As expected, those variants that are more common in non-African populations are found in higher proportions in both of the Neanderthal genomes studied here, with a slightly higher proportion for the Vindija genome, which is in fact assumed to be closer to the main source population of introgression^33^. We detect a smaller contribution of Denisovan variants overall, which is expected on several grounds: given the likely more frequent interactions between modern humans and Neanderthals, the Denisovan individual whose genome we relied on is likely part of a more pronounced “outgroup”. Gene flow from modern humans into Neanderthals also likely contributed to this pattern.

In the case of the regions under putative positive selection, we find that the distribution of variant counts has a local peak in the most recent period (0–100 kya) that is absent from the deserts of introgression datasets, pointing to an earlier origin of alleles found in these latter regions. Also, as shown in Fig. 2d, the distribution of variant counts in these regions under selection shows the greatest difference between the two peaks of the bimodal distribution. Still, we should stress that our focus here is on HF variants, and that of course, not all HF variants falling in selective sweep regions were actual targets of selection. Figure S5 illustrates this point for two genes that have figured prominently in early discussions of selective sweeps since^5^: *RUNX2* and *GLI3*. While recent HF variants are associated with positive selection signals (indicated in purple), older variants exhibit such associations as well. Indeed some of these targets may fall below the 90% cutoff chosen in^19^. In addition, we are aware that variants enter the genome at one stage and are likely selected for at a (much) later stage^34,35^. As such our study differs from the chronological atlas of natural selection in our species presented in^36^ (as well as from other studies focusing on more recent periods of our evolutionary history, such as^37^). This may explain some important discrepancies between the overall temporal profile of genes highlighted in^36^ and the distribution of HF variants for these genes in our data (Fig. S6).

Having said this, our analysis recaptures earlier observations about prominent selected variants, located around the most recent peak, concerning genes such as *CADPS2*^38^ (Fig. S7). This study also identifies a set of old variants, well before 300kya, associated with genes belonging to putative positively-selected regions before the deepest divergence of *Homo sapiens* populations^39^, such as *LPHN3*, *FBXW7*, and *COG5* (Fig. S8).

Finally, focusing on the brain as the organ that may help explain key features of the rich behavioral repertoire associated with *Homo sapiens*, we estimated the age of putative regulatory variants linked to the prefrontal (PFC), temporal (TC), and cerebellar cortices (CBC), using the large scale characterization of regulatory elements of the human brain provided by the PsychENCODE Consortium^40^. We did the same for the modern human HF missense mutations^19^. A comparative plot reveals a similar pattern between the three structures, with no obvious differences in variant distribution (see Fig. S9). The cerebellum contains a slightly higher number of variants assigned to the more recent peak when the proportion to total mapped variants is computed. This may relate to the more recent modifications reported for this brain region^41^, which contributed to the globularized shape of our brain(case). We also note that the difference of dated variants between the two local maxima is more pronounced in the case of the cerebellum than in the case of the two cortical tissues, whereas this difference is more reduced in the case of missense variants (Fig. S9). We caution, though, that the overall number of missense variants is considerably lower in comparison to the other three datasets.

### Gene Ontology analysis across temporal windows

**Figure 3:**
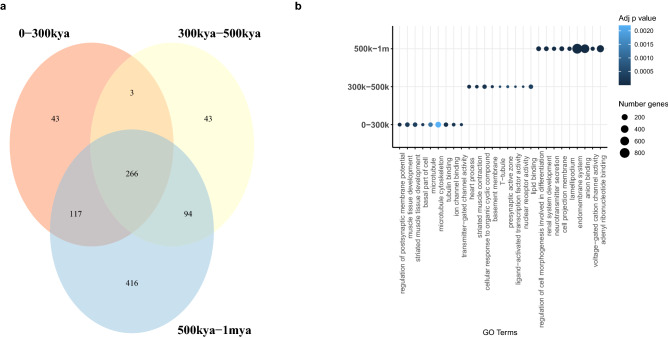
(**a**) Venn diagram of GO terms associated with genes shared across time windows. (**b**) Top GO terms per time window.

In order to interpret functionally the distribution of HF variants in time, we performed enrichment analyses accessing curated databases via the *gProfiler2* R package^42^. For the three time windows analyzed (corresponding to the recent peak: 0–300 kya; divergence time and earlier peak: 500 kya–1 mya; and time slot between them: 300 kya–500 kya), we identified unique and shared gene ontology terms (see Fig. 3a,b; “Methods”). Notably, when we compared the most recent period against the two earlier windows together (from 300 kya to 1 mya), we found bone, cartilage, and visual system-related terms only in the earlier periods (hypergeometric test; adj. $p<0.01$; Table S1). Further differences are observed when thresholding by an adjusted $p<0.05$. In particular, terms related to behavior (startle response), facial shape (narrow mouth) and hormone systems only appear in the middle (300–500 k) period (Table S2; Fig. S10). Unique gene ontology terms may point to specific environmental conditions causing the organism to react in specific ways. A summary of terms shared across the three time windows can be seen in Fig. S11.

### Gene expression predictions

**Figure 4:**
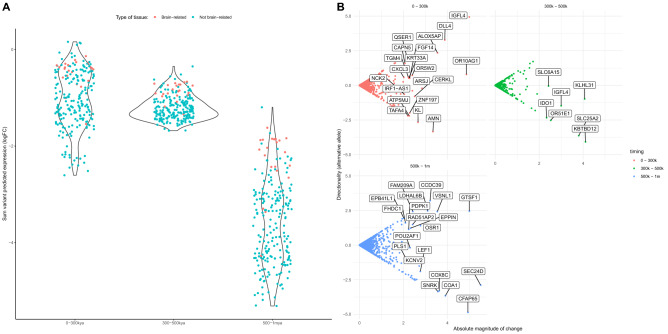
(**A**) Sum of all directional mutation effects within 1 kb to the TSS per time window in 22 brain and brain-related tisues (red) and the the rest of tissues included by the ExPecto trained model as a control group (blue). Significant differences exist across time periods when non-brain and brain-related tissues are compared (Kruskal–Wallis test; $p=2.2e-16$). (**B**) Genes with a high sum of all directional mutation effects, and cumulative directionality of expression values in brain tissues per time window.

To evaluate the expression profiles associated to our HF variant dataset (from^19^), we made use of ExPecto^21^, a sequence-based tool to predict gene expression *in silico* (see description in “Methods”). We found a skewness towards more extreme negative values (downregulation) in brain-related tissues, which is not observed when analyzing all tissues jointly (as shown in quantile-quantile plots in Fig. S12). A series of Kruskal-Wallis test shows that, when either all or just brain-related tissues are considered, statistically significant differences in predicted gene expression values are found across the three time periods studied here (p = 2.2e−16 and p = 4.95e−12, respectively). Overall, the latest period (500 k–1 mya) reports the strongest predicted effect toward downregulation (see Fig. 4A). Especially for brain-related terms, some structures show the highest sum of variant predicted expression (top downregulation): such as the Adrenal Gland, the Pituitary, Astrocytes, or Neural Progenitor Cells (see Fig. S13). Among these structures, the presence of the cerebellum in a period preceding the last major Out-of-Africa event is noteworthy (consistent with^41^).

The authors of the article describing the ExPecto tool^21^ suggest that genes with a high sum of absolute variant effects in specific time windows tend to be tissue or condition-specific. We explored our data to see if the genes with higher absolute variant effect were also phenotypically relevant (Fig. 4B). Among these we find genes such as *DLL4*, a Notch ligand implicated in arterial formation^43^; *FGF14*, which regulates the intrinsic excitability of cerebellar Purkinje neurons^44^; *SLC6A15*, a gene that modulates stress vulnerability through the glutamate system^45^; and *OPRM1*, a modulator of the dopamine system that harbors a HF derived loss of stop codon variant in the genetic pool of modern humans but not in that of extinct human species^19^.

We also crosschecked if any of the variants in our high-frequency dataset with a high predicted expression value (RPKM variant-specific values at $log>0.01$) were found in GWASs related to brain volume. The Big40 UKBiobank GWAS meta-analysis^46^ shows that some of these variants are indeed GWAS top hits and can be assigned a date (see Table 1). Of note are phenotypes associated with the posterior Corpus Callosum (Splenium), precuneus, and cerebellar volume. In addition, in a large genome-wide association meta-analysis of brain magnetic resonance imaging data from 51,665 individuals seeking to identify specific genetic loci that influence human cortical structure^47^, one variant (rs75255901) in Table 1, linked to *DAAM1*, has been identified as a putative causal variant affecting the precuneus. All these brain structures have been independently argued to have undergone recent evolution in our lineage^41,48–50^, and their associated variants are dated amongst the most recent ones in the table.

**Table 1:** Big40 Brain volume GWAS^46^ top hits with high predicted gene expression in ExPecto ($log>0.01$, RPKM), along with dating as provided by *GEVA*.

Location	rsid	Nearest gene(s)	GWAS trait	Age (GEVA)
20:49070644	rs75994450	PTPN1	Fractional anisotropy measurement, Splenium (Corpus Callosum)	36,735.46
14:59669037	rs75255901	DAAM1	Functional connectivity (rfMRI)	39,543.24
1:22498451	rs2807369	WNT4	Volume of gray matter in Cerebellum (left)	50,060.96
2:63144695	rs17432559	EHBP1	Volume of Corpus Callosum (Posterior)	52,290.48
12:2231744	rs75557252	CACNA1C	Functional connectivity (rfMRI)	93,924.62
10:92873811	rs17105731	PCGF5	Volume of inferiortemporal gyrus (right)	255,792.5
17:59312894	rs73326893	BCAS3	Functional connectivity (rfMRI)	418,742.6
22:27195261	rs72617274	CRYBA4	Functional connectivity (rfMRI)	445,477.7
2:230367803	rs56049535	DNER	Functional connectivity (rfMRI)	523,629.8
16:3687973	rs78315731	DNASE1	Volume of Pars triangularis (left)	698,856.5

‘Functional connectivity’ is a measure of temporal activity synchronization between brain parcels at rest (originally defined in^51^).

## Discussion

Deploying GEVA to probe the temporal structure of the extended catalog of HF variants distinguishing modern humans from their closest extinct relatives ultimately aims to contribute to the goals of the emerging attempts to construct a molecular archaeology^52^ and as detailed a map as possible of the evolutionary history of our species^53^. Like any other archaeology dataset, ours is necessarily fragmentary. In particular, fully fixed mutations, which have featured prominently in early attempts to identify candidates with important functional consequences^52^, fell outside the scope of this study, as GEVA can only determine the age of polymorphic mutations in the present-day human population. By contrast, the mapping of HF variants was reasonably good, and allowed us to provide complementary evidence for claims regarding important stages in the evolution of our lineage. This in and of itself reinforces the rationale of paying close attention to an extended catalog of HF variants, as argued in^19^.

While we wait for more genomes from more diverse regions of the planet and from a wider range of time points, we find our results encouraging: even in the absence of genomes from the deep past of our species in Africa, we were able to provide evidence for different epochs and classes of variants that define these. But whereas different clusters can be identified, the emerging picture is very much mosaic-like in its character, in consonance with recent work^1,3^. In no way do we find evidence for earlier evolutionary narratives that relied on one or a handful of key mutations.

Our analysis shows a bimodal distribution of the age of modern human-derived high-frequency variants (in consonance with the findings of^54^ on a more limited set of variants ). The two peaks likely reflect, on the one hand, the point of divergence between *Homo sapiens* and other *Homo* species and, on the other, the period of population dispersal and replacement following the last major out of Africa event.

Our work also highlights the importance of a temporal window right before 300 ky that may well correspond to a significant behavioral shift in our lineage, such as increased ecological resource variability^55^, and evidence of long-distance stone transport and pigment use^56^. Other aspects of our cognitive and anatomical make up emerged much more recently, in the last 150 k years, and for these our analysis points to the relevance of gene expression regulation differences in recent human evolution, in line with^57–59^.

Lastly, our attempt to date the emergence of mutations in our genomes points to multiple episodes of introgression, whose history is likely to turn out to be quite complex.

## Methods

### Homo sapiens variant catalog

We made use of a publicly available dataset^19^ that takes advantage of the Neanderthal and Denisovan genomes to compile a genome-wide catalog of *Homo sapiens*-specific variation. The original complete dataset is available at https://doi.org/10.6084/m9.figshare.8184038. As described in the original article, this catalog includes “archaic”-specific variants and all loci showing variation within modern populations. The 1000 genomes project and ExAc data were used to derive frequencies and the human genome version *hg19* as reference. As indicated in the original publication^19^, quality filters in the “archaic” genomes were applied (specifically: sites with less 5-fold coverage and more than 105-fold coverage for the Altai individual, or 75-fold coverage for the rest of “archaic” individuals were filtered out). In ambiguous cases, variant ancestrality was determined using multiple genome aligments^60^ and the macaque reference sequence (*rheMac3*)^61^.

In addition to the full data, the authors offered a subset of the data that includes derived variants at a $\ge$ 90% global frequency cutoff. Since such a cutoff allows some variants to reach less than 90% in certain populations, as long as the total is $\ge$ 90%, we also considered including a metapopulation-wide variant $\ge$ 90% frequency cutoff dataset to this study (Fig. S2). All files (including the original full and high-frequency sets and the modified, stricter high-frequency one) are provided in the accompanying code. Controls in 1 were obtained through a probabilistic permutation approach with sets of random variants (100 sets, 50,000 variants each).

### GEVA

The Genealogical Estimation of Variant Age (GEVA) tool^20^ uses a hidden Markov model approach to infer the location of ancestral haplotypes relative to a given variant. It then infers time to the most recent ancestor in multiple pairwise comparisons by coalescent-based clock models. The resulting pairwise information is combined in a posterior probability measure of variant age. We extracted dating information for the alleles of our dataset from the bulk summary information of GEVA age predictions. The GEVA tool provides several clock models and measures for variant age. We chose the mean age measure from the joint clock model, that combines recombination and mutation estimates. While the GEVA dataset provides data for the 1000 genomes project and the Simons Genome Diversity Project, we chose to extract only those variants that were present in both datasets. Ensuring a variant is present in both databases implicitly increases genealogical estimates (as detailed in Supplementary document 3 of^20^), although it decreases the amount of sites that can be looked at. We give estimated dates after assuming 29 years per generation, as suggested in^62^. While other measures can be chosen, this value should not affect the nature of the variant age distribution nor our conclusions.

Out of a total of 4,437,804 for our total set of variants, 2,294,023 where mapped in the GEVA dataset (51% of the original total). For the HF subsets, the mapping improves: 101,417 (74% of total) and 48,424 (69%) variants were mapped for the original high-frequency subset and the stricter, meta-population cutoff version, respectively.

### ExPecto

In order to predict gene expression we made use of the *ExPecto* tool^21^. *ExPecto* is a deep convolutional network framework that predicts tissue-specific gene expression directly from genetic sequences. *ExPecto* is trained on histone mark, transcription factor and DNA accessibility profiles, allowing ab initio prediction that does not rely on variant information training. Sequence-based approaches, such as the one used by *Expecto*, allow to predict the expression of high-frequency and rare alleles without the biases that other frameworks based on variant information might introduce. We introduced the high-frequency dated variants as input for *ExPecto* expression prediction, using the default tissue training models trained on the GTEx, Roadmap genomics and ENCODE tissue expression profiles.

### gProfiler2

Enrichment analysis was performed using *gProfiler2* package^42^ (hypergeometric test; multiple comparison correction, ‘gSCS’ method; p values 0.01 and 0.05). Dated variants were subdivided in three time windows (0–300 kya, 300–500 kya and 500 kya–1 mya) and variant-associated genes (retrieved from^19^) were used as input (all annotated genes for *H. sapiens* in the Ensembl database were used as background). Following^21^, variation potential directionality scores were calculated as the sum of all variant effects in a range of 1 kb from the TSS. Summary GO figures presented in Fig. S11 were prepared with *GO Figure*^63^.

For enrichment analysis, the Hallmark curated annotated sets^64^ were also consulted, but the dated set of HF variants as a whole did not return any specific enrichment.

## Supplementary Information

Supplementary Information 1.

Supplementary Information 2.

Supplementary Information 3.

## Data Availability

All the analysis here presented can be reproduced following the scripts in the following Github repository: https://github.com/AGMAndirko/Temporal-mapping.
