# Supplementary material for: Temporal mapping of derived high-frequency gene variants supports the mosaic nature of the evolution of Homo sapiens
Source: Sci Rep. 2022 Jun 15;12:9937. doi: 10.1038/s41598-022-13589-0 (PMC9200848; doi:10.1038/s41598-022-13589-0)
Supplement: Supplementary file 1 — Supplementary Information 1. [file 41598_2022_13589_MOESM1_ESM.pdf]

Supplementary Figures

for

"Temporal mapping of derived high-frequency gene variants supports the mosaic nature of the evolution of *Homo sapiens*"

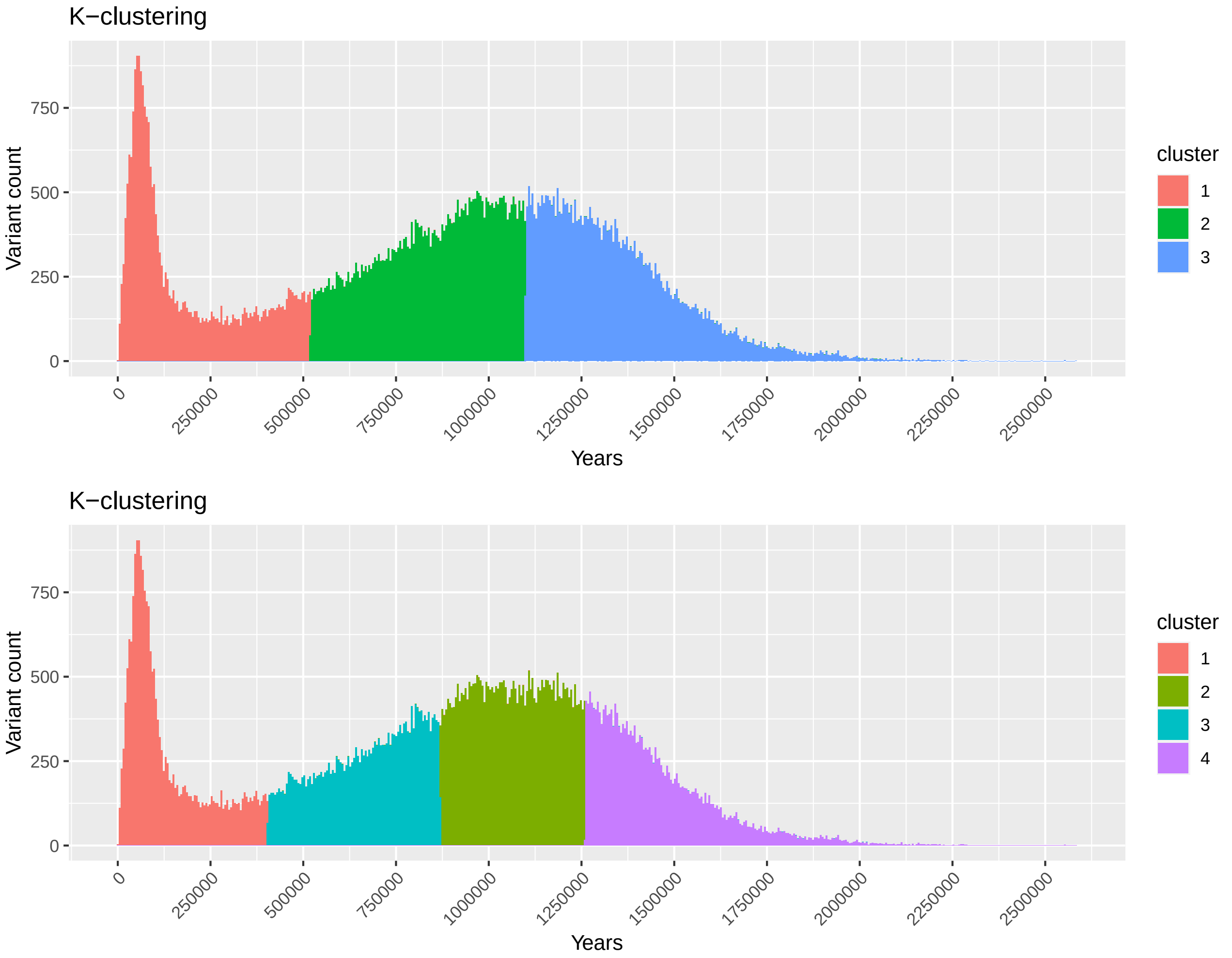

Figure 1: K -means clustering analysis of HF variant temporal distribution, for both k = 3 (top) and k = 4 (bottom).

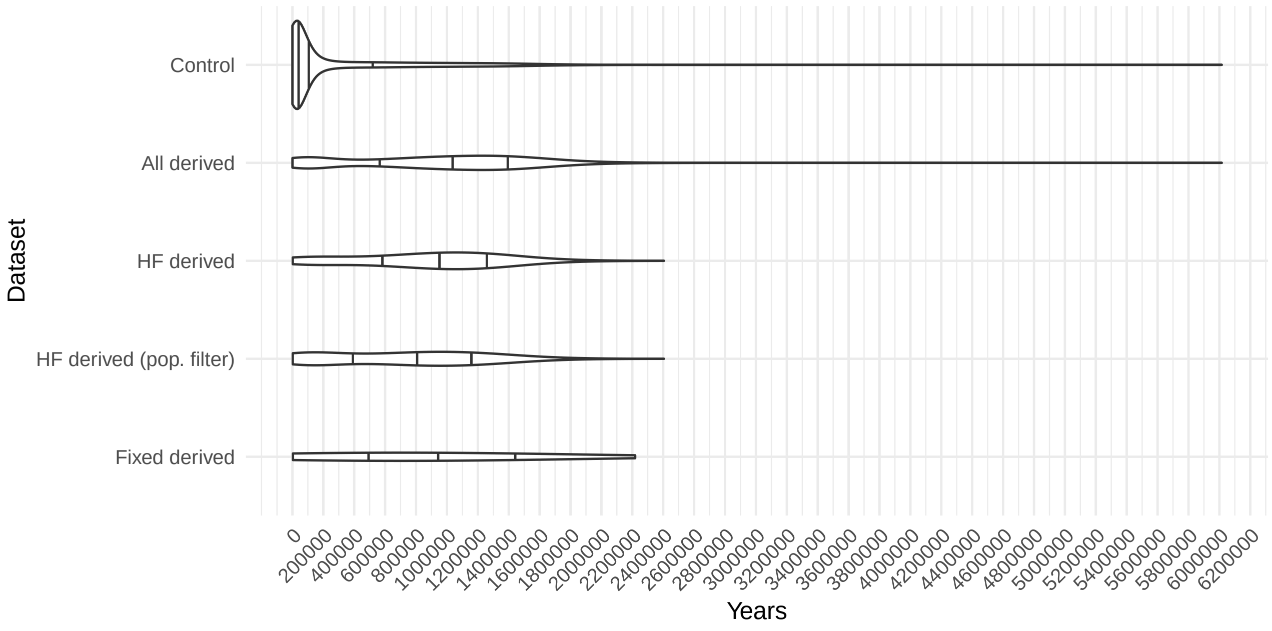

Figure 2: Density distribution of derived *Homo sapiens* alleles for different subsets used in this study (related to Figure 1). From top to bottom, control set of random variants; all derived variants in the *Homo sapiens* lineage; those variants at high-frequency (HF); HF variants with an added meta-population filter (see sec. 4); and variants that reached fixation. A direct comparison of all variants, HF and HF with the added filter can be found in Figure S2. Horizontal lines mark distribution quantiles 0.25, 0.5 and 0.75.

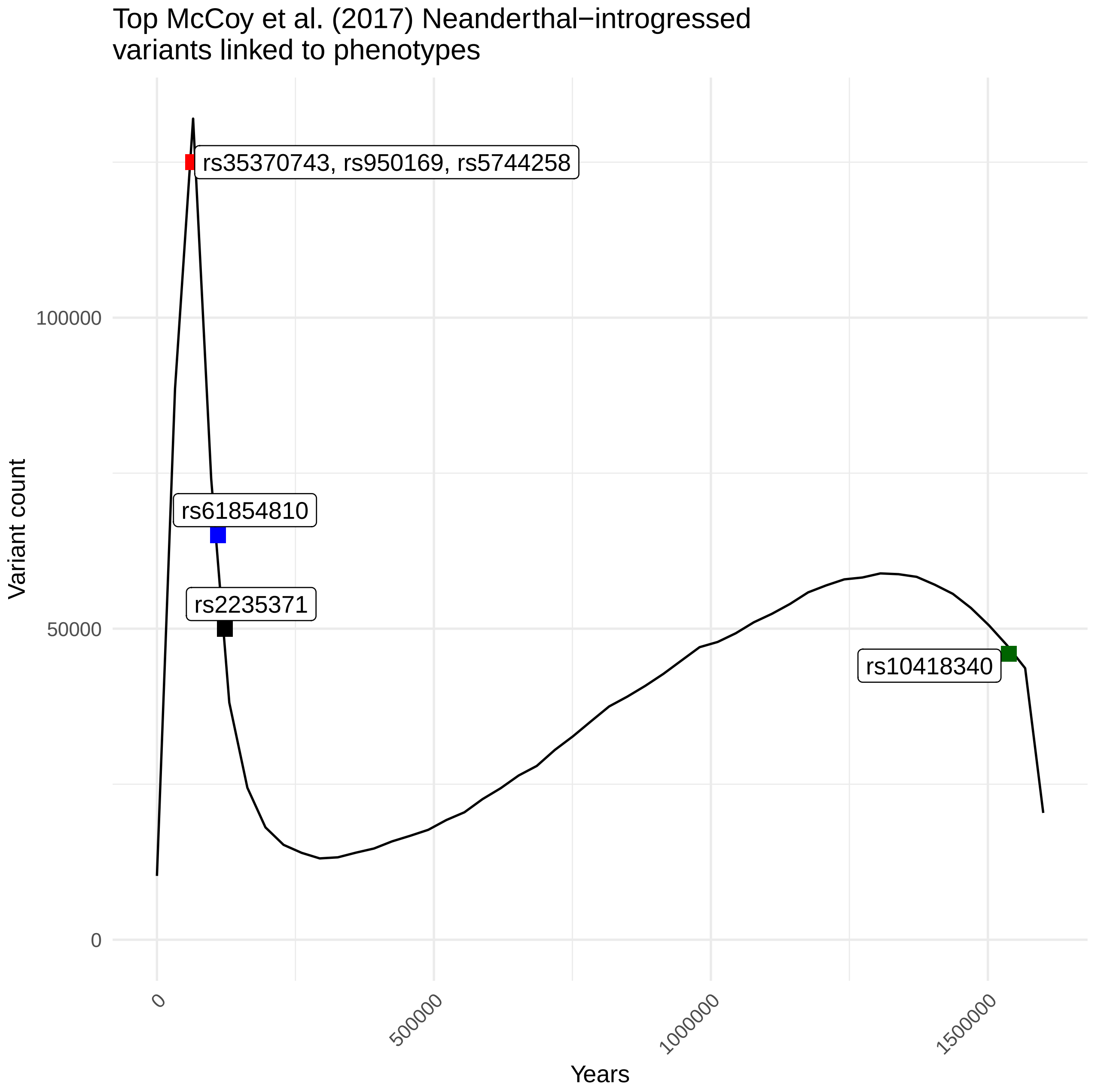

Figure 3: Temporal distribution of introgressed variants linked to phenotypes, as highlighted in Table 1 of [1], compared to the distribution of all derived variants over time.

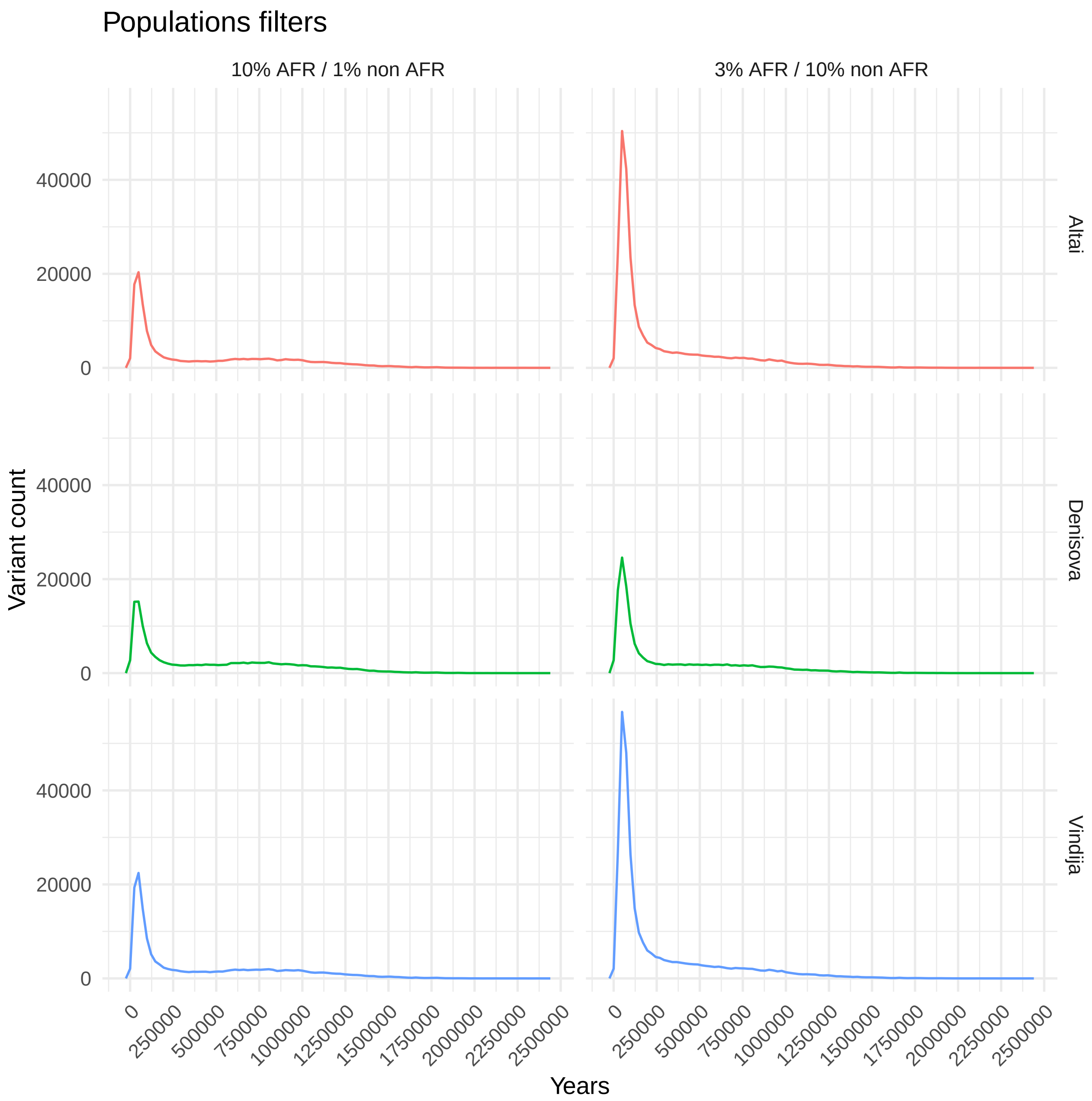

Figure 4: Temporal distribution of variants shared with each of the extinct human genomes after applying specific population frequency filters. These filter include a 10% minor allele frequency cutoff in the African metapopulation (AFR), coupled with a 1% cutoff in the rest of metapopulations, designed to detect potential introgressed alleles brought into the African genetic pool by back-to-Africa migration events. The second filter applied is a 3% cutoff in AFR populations and a 10% threshold in non-African populations, designed to detect the contribution of each extinct human sample to the introgresed variant genetic pool, accounting for a third of that pool to be introduced in AFR populations by back-to-Africa migrations.

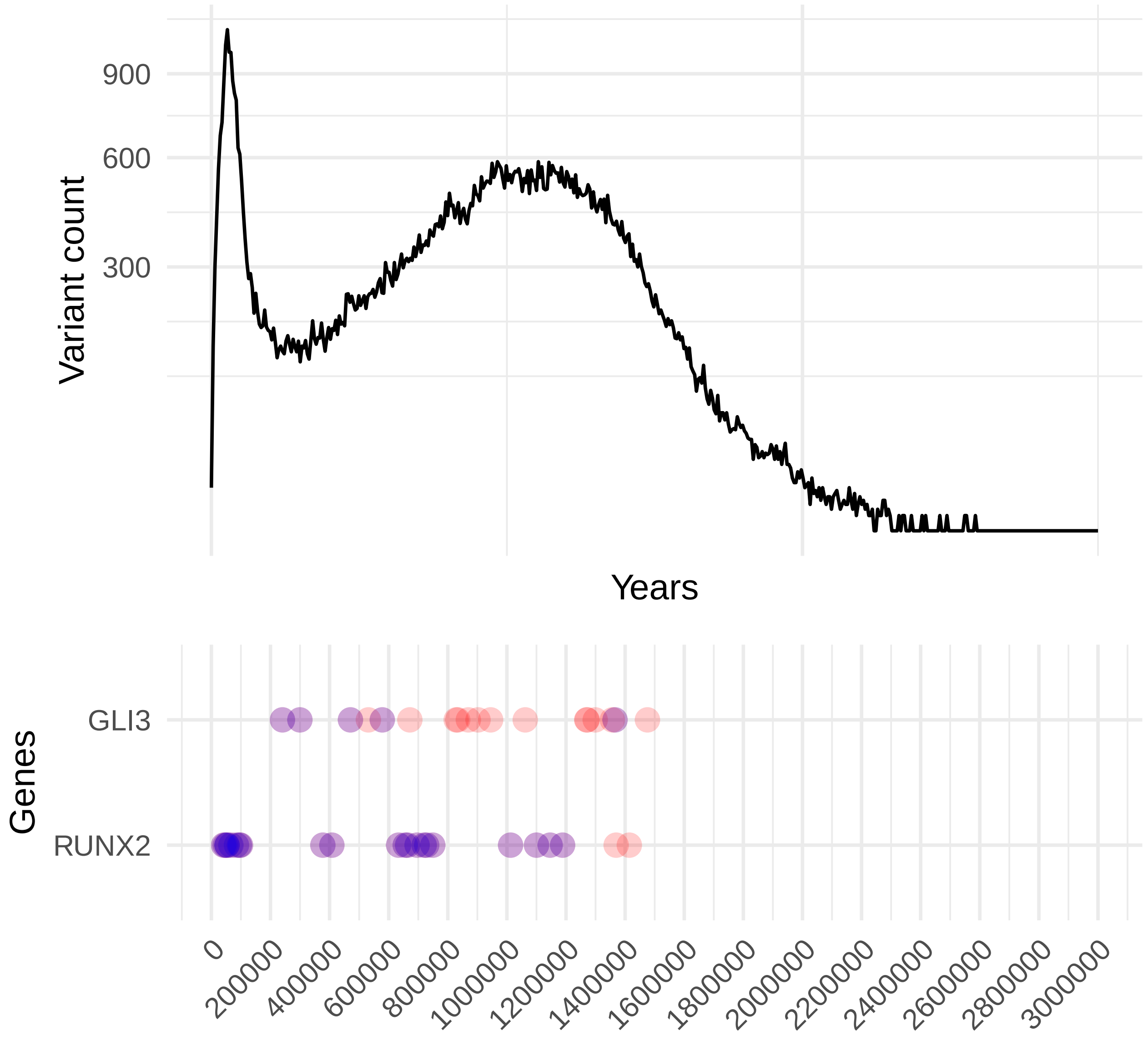

Figure 5: Temporal distribution of HF variants in two genes highlighted in early discussions of selective sweeps: *GLI3* (sweep region from [2]) and *RUNX2* (sweep region from [3]). Variants in purple fall within sweep regions.

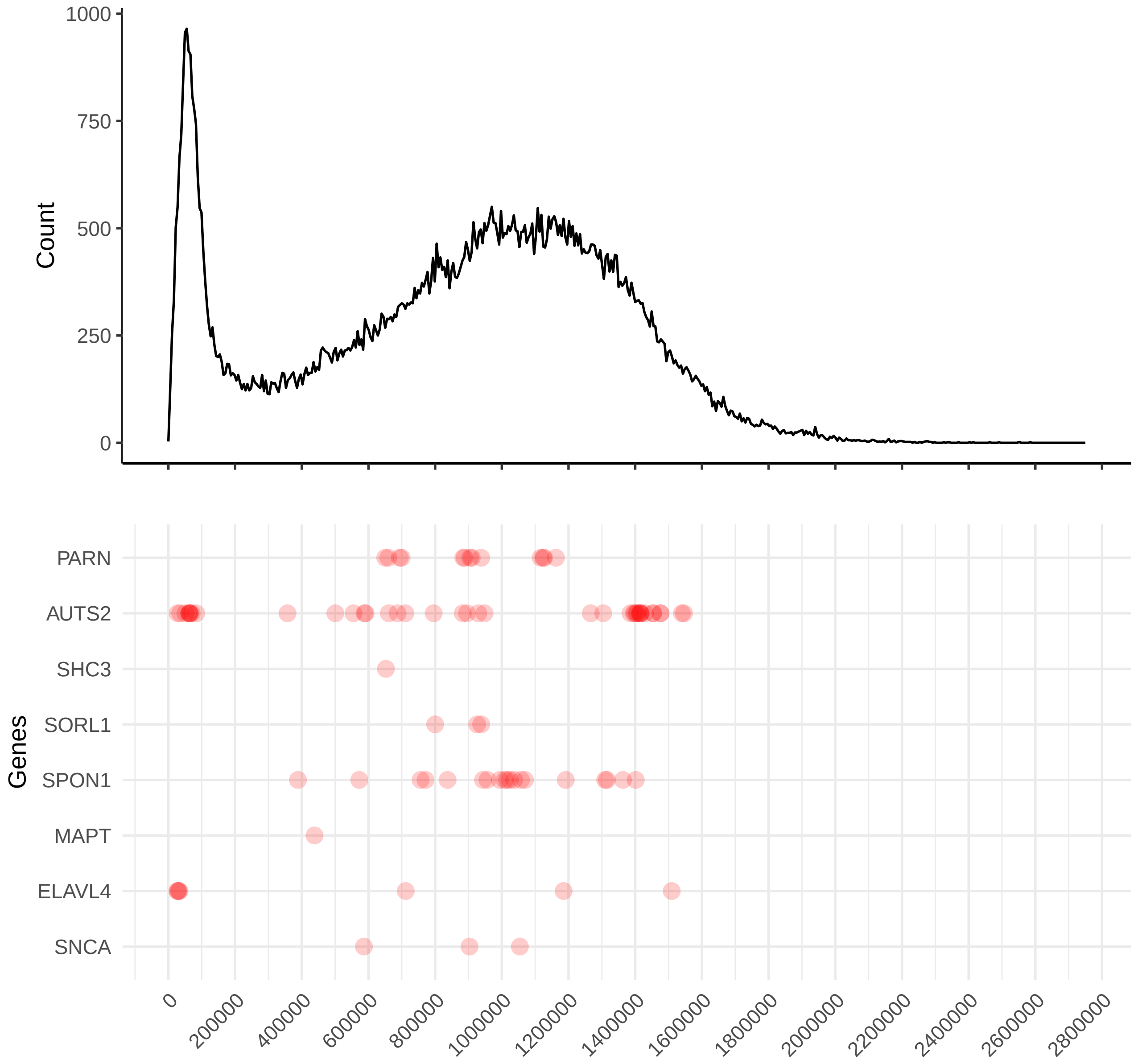

Figure 6: Temporal distribution of variants associated with genes highlighted in [4].

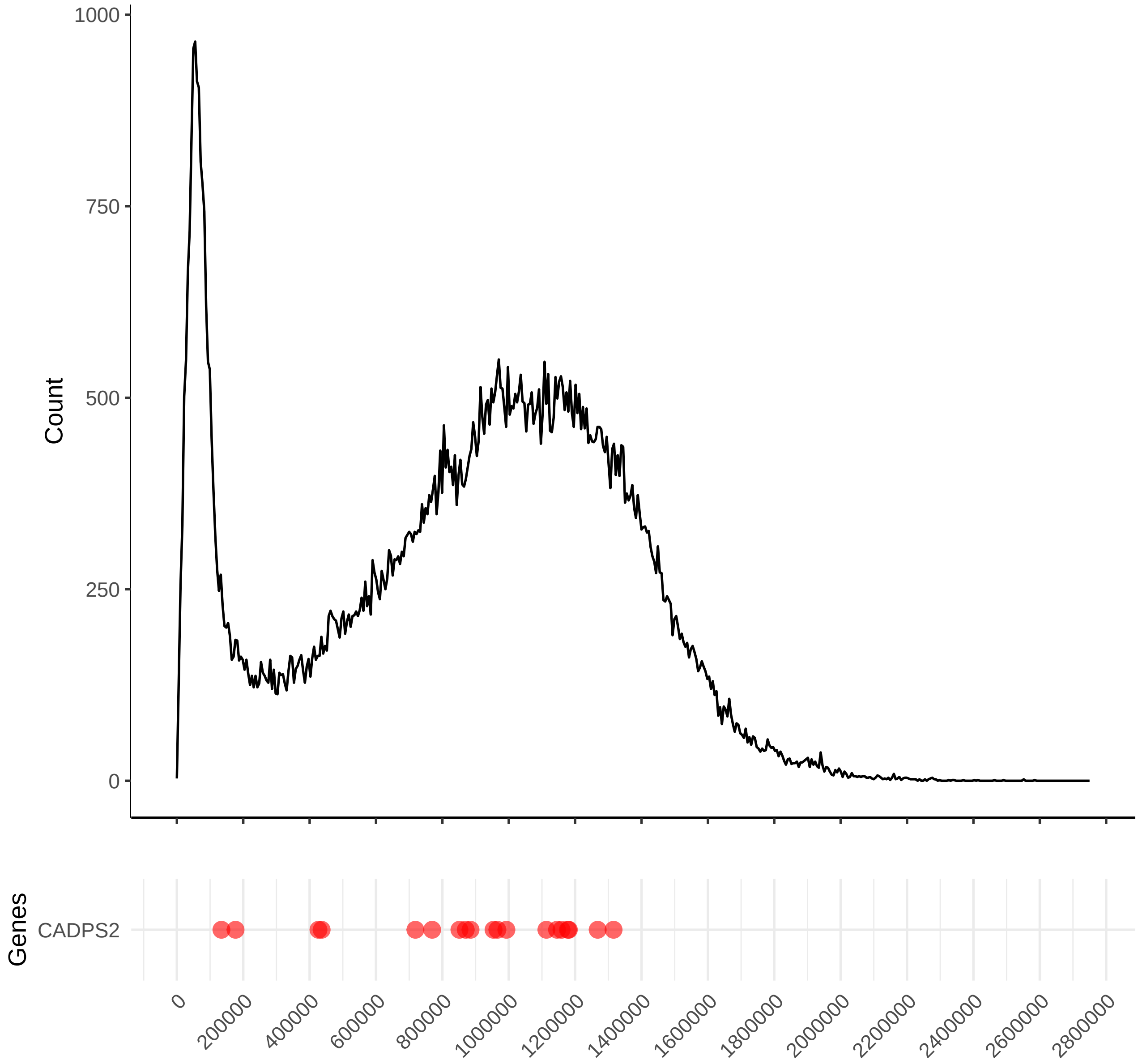

Figure 7: Temporal distribution of variants associated with *CADPS2*. The most recent variants around 200kya in particular capture the reasons this gene was highlighted in [5]: "*CADPS2* was identified in [3] as a candidate for selection . . . . The gene has been suggested to be specifically important in the evolution of all modern humans, as it was not found to be selected earlier in great apes or later in particular modern human populations".

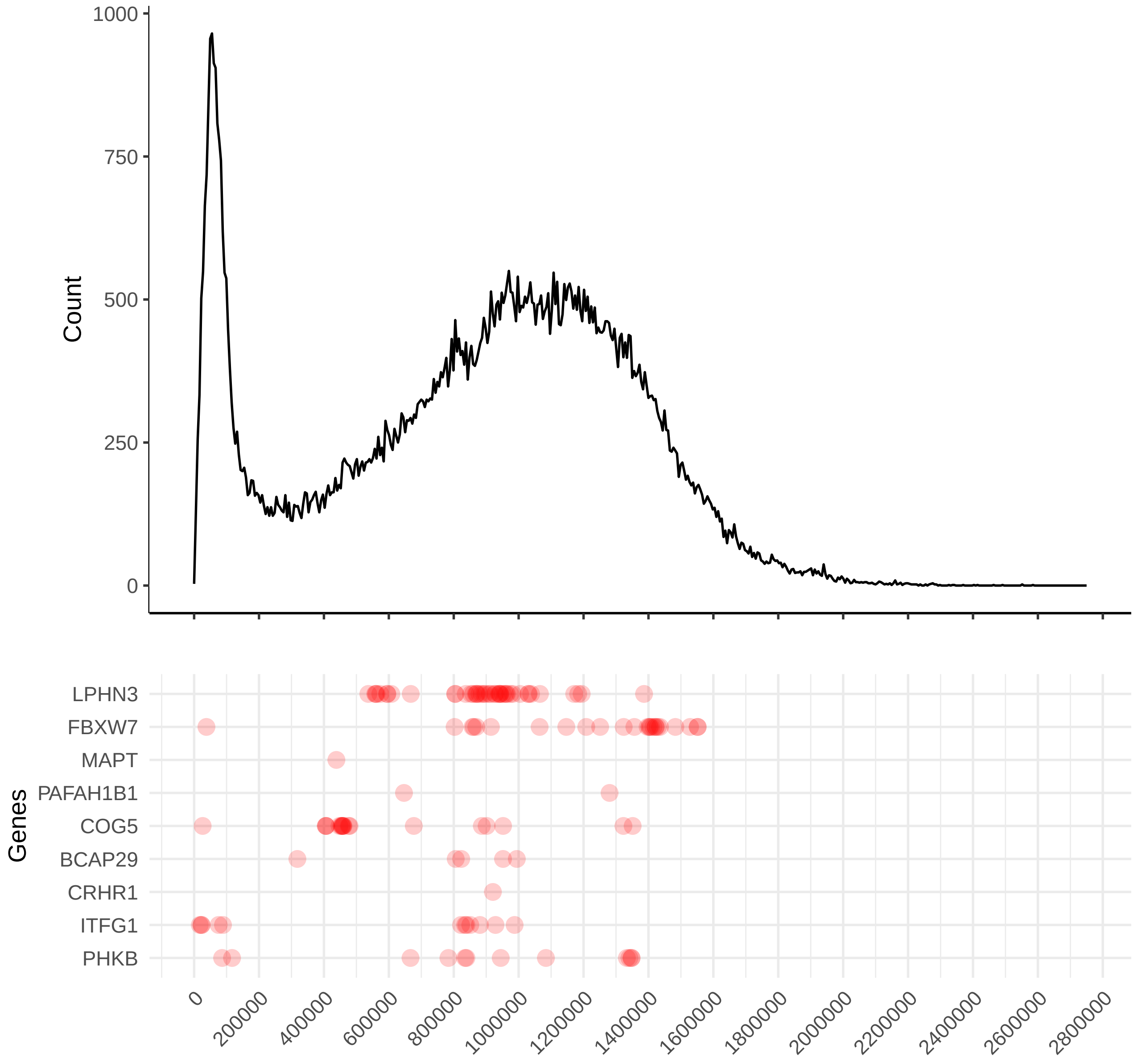

Figure 8: Temporal distribution of variants in genes found in putative positively-selected genetic windows before early *Homo sapiens* population divergence, as per [6]. Genes belonging to putative positively selected regions were retrieved from Supplementary Data, section 12 of [6].

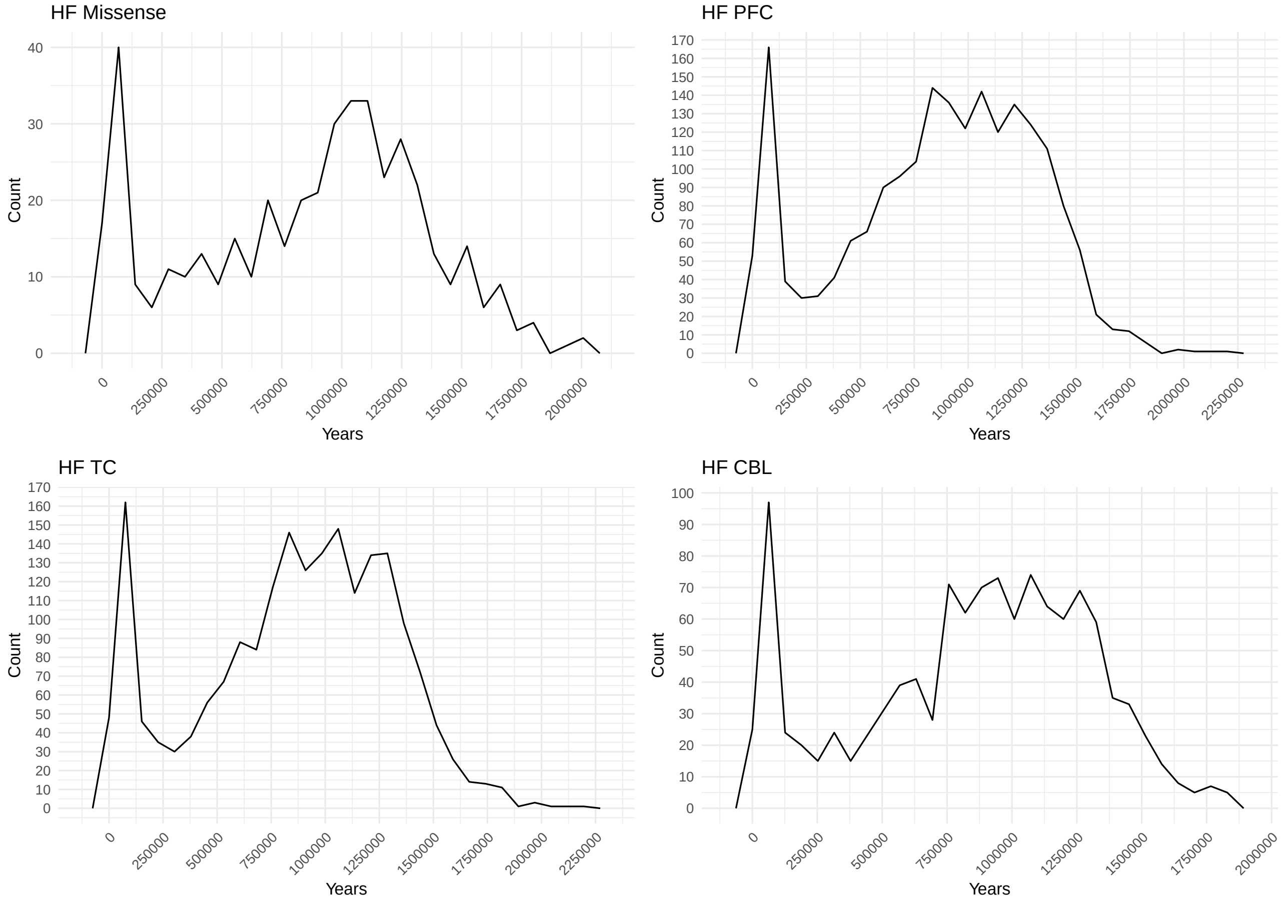

Figure 9: Temporal distribution of high-frequency missense and regulatory variants. Missense variants derived from [7]; enhancer annotations for the prefrontal, temporal and cerebellar cortices were retrieved from [8]. The difference between the two total maximum counts in the left to the right peak is more pronounced in the cerebellum and prefrontal cortices (23 and 22 more variants mapped to the left maximum peak, respectively). This same difference for missense variants is reduced to only 7 more variants mapped to the left maximum peak. For the temporal cortex, this difference amounts to 14 mapped variants.

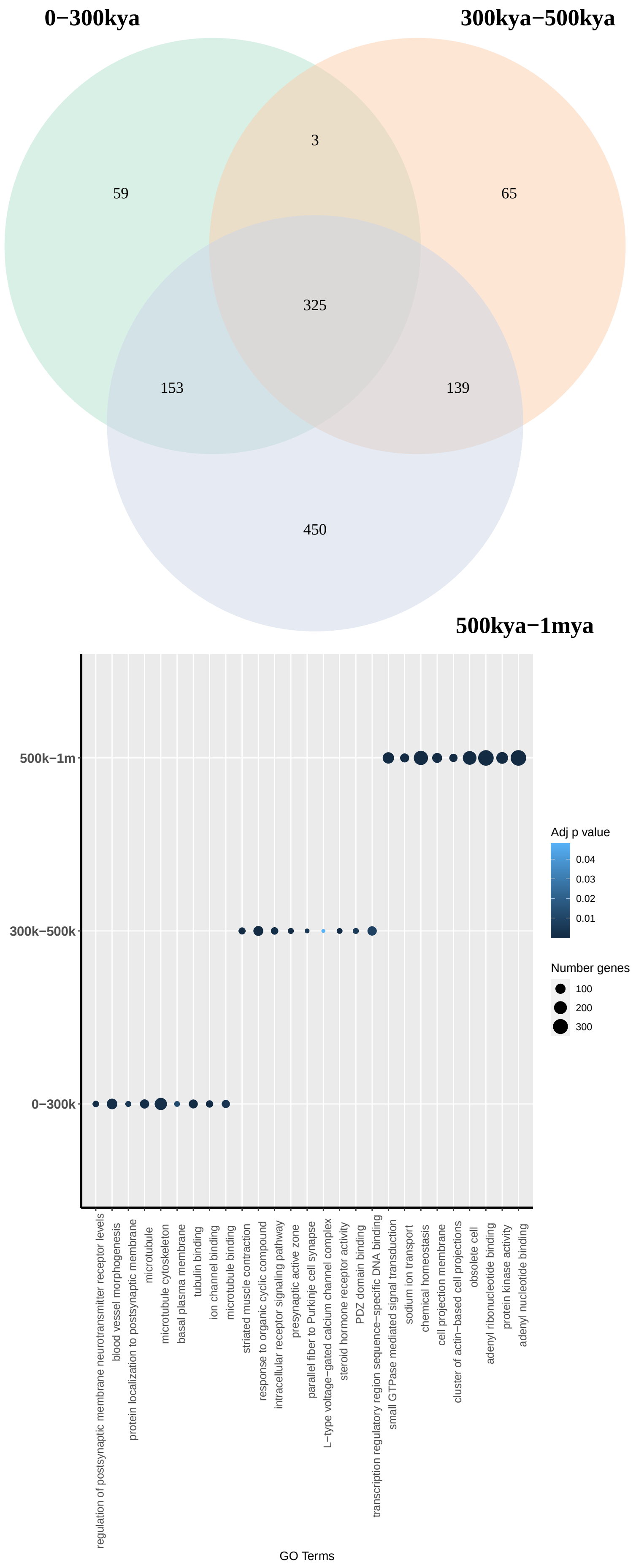

Figure 10: GO terms results when thresholding by an adjusted p-value of 0.05. Venn diagram (top) shows number of unique and shared GO terms across periods. Dot plot (bottom) highlights the top 3 GO terms by significance for each period.

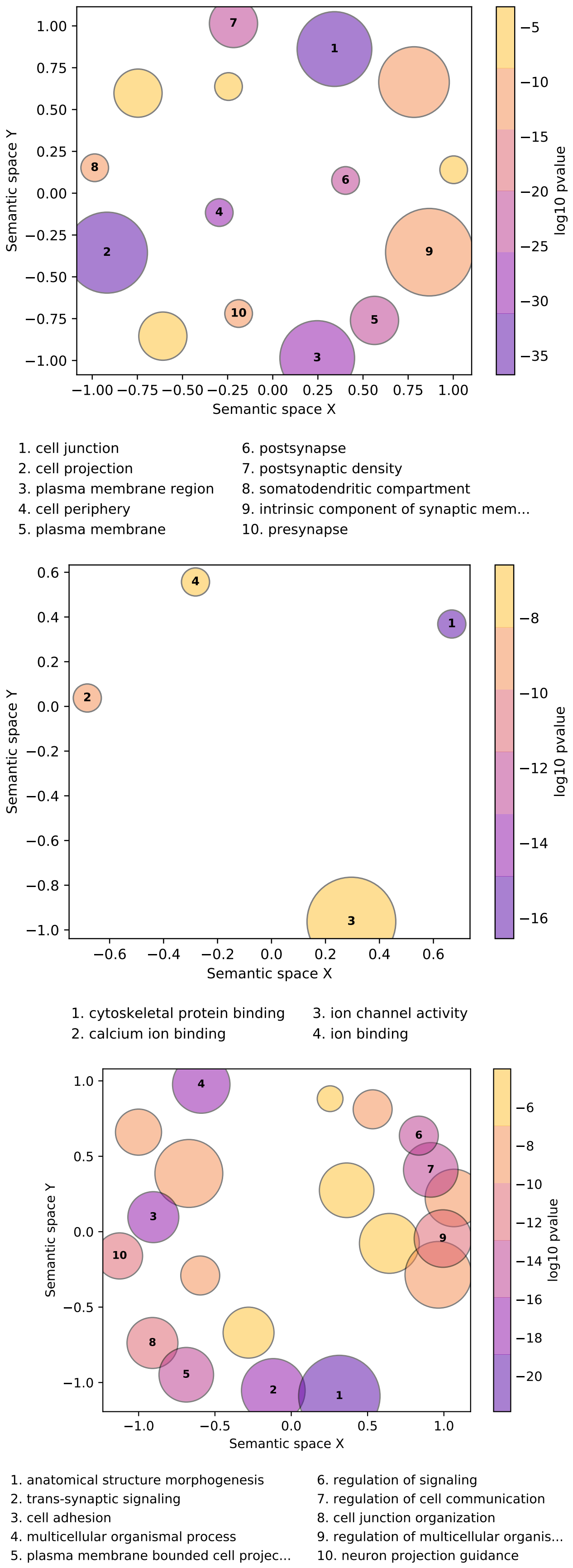

Figure 11: GO term reduction of shared terms across time windows (center of Venn diagram in Fig. 3A).

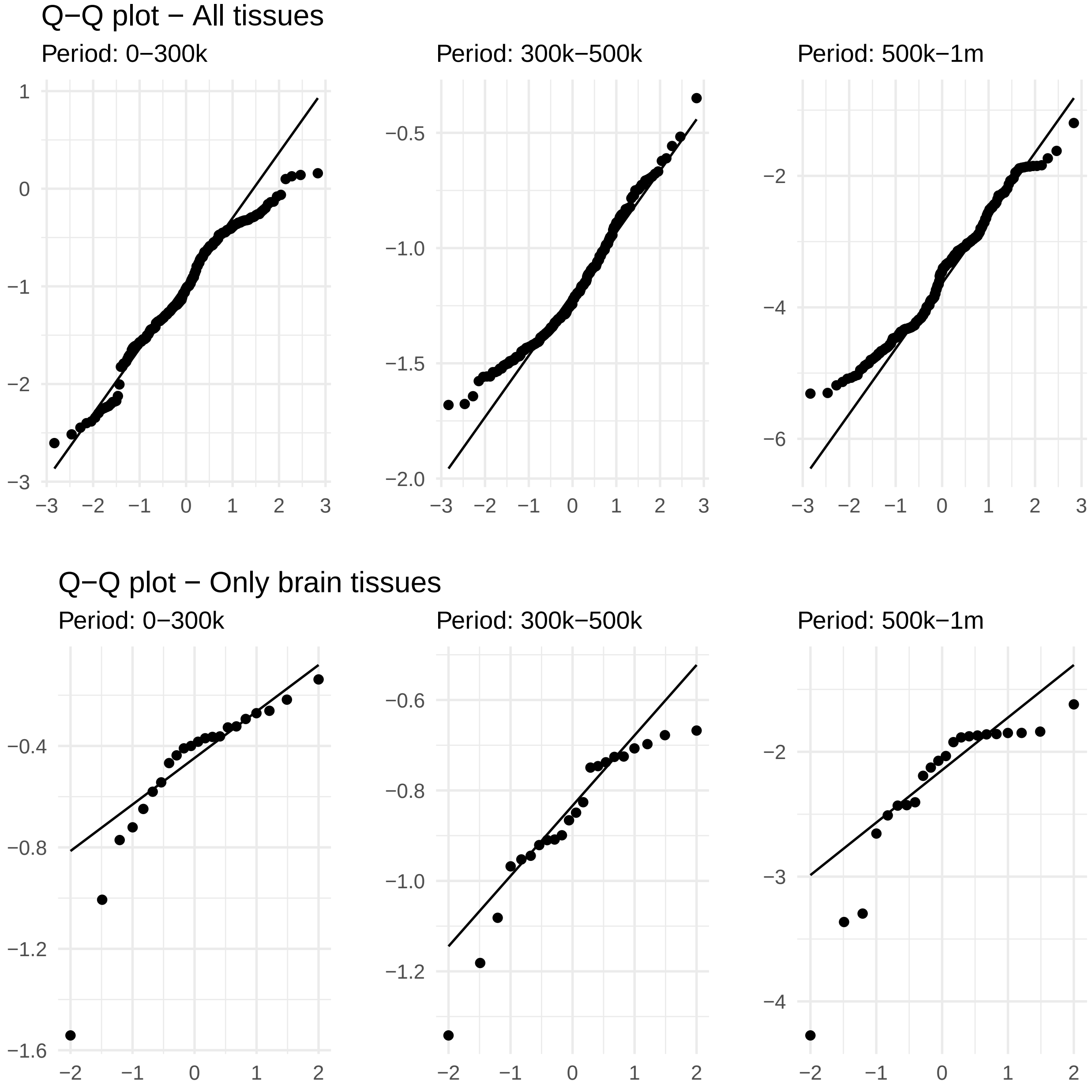

Figure 12: Quantile-quantile plots of predicted expression values of high-frequency variants, divided in three time periods (0-300kya, 300k-500kya and 500k-1mya). Applied to both all tissues and only brain-related tissues.

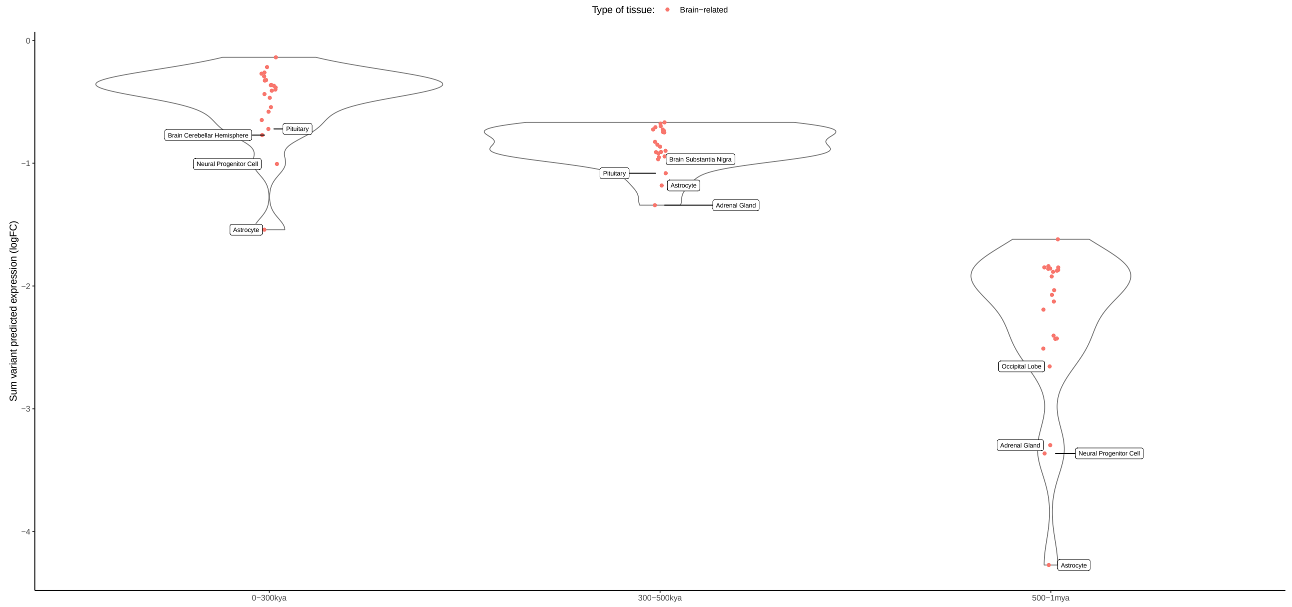

Figure 13: Violin plots per time window for 22 brain and brain-related tissues, showing the top-4 structures with strongest predicted downregulation.

References

[1] McCoy, R. C., Wakefield, J. & Akey, J. M. Impacts of Neanderthal-Introgressed Sequences on the Landscape of Human Gene Expression. *Cell* **168**, 916–927.e12, DOI: 10.1016/j.cell.2017.01.038 (2017).

[2] Peyrégne, S., Boyle, M. J., Dannemann, M. & Prüfer, K. Detecting ancient positive selection in humans using extended lineage sorting. *Genome Research* **27**, 1563–1572, DOI: 10.1101/gr.219493.116 (2017).

[3] Green, R. E. *et al.* A Draft Sequence of the Neandertal Genome. *Science* **328**, 710–722, DOI: 10.1126/science.1188021 (2010).

[4] Zhou, H. *et al.* A Chronological Atlas of Natural Selection in the Human Genome during the Past Half-million Years. *bioRxiv* 018929, DOI: 10.1101/018929 (2015).

[5] Racimo, F. Testing for Ancient Selection Using Cross-population Allele Frequency Differentiation. *Genetics* **202**, 733–750, DOI: 10.1534/genetics.115.178095 (2016).

[6] Schlebusch, C. M. *et al.* Khoe-San Genomes Reveal Unique Variation and Confirm the Deepest Population Divergence in Homo sapiens. *Molecular Biology and Evolution* **37**, 2944–2954, DOI: 10.1093/molbev/msaa140 (2020).

[7] Kuhlwilm, M. & Boeckx, C. A catalog of single nucleotide changes distinguishing modern humans from archaic hominins. *Scientific Reports* **9**, 8463, DOI: 10.1038/s41598-019-44877-x (2019).

[8] Wang, D. *et al.* Comprehensive functional genomic resource and integrative model for the human brain. *Science* **362**, eaat8464, DOI: 10.1126/science.aat8464 (2018).
